# The Herpes Simplex Virus Neurovirulence Factor γ34.5: Revealing Virus–Host Interactions

**DOI:** 10.1371/journal.ppat.1005449

**Published:** 2016-03-10

**Authors:** Douglas R. Wilcox, Richard Longnecker

**Affiliations:** Department of Microbiology and Immunology, Northwestern University Feinberg School of Medicine, Chicago, Illinois, United States of America; Columbia University, UNITED STATES

## Introduction

**Fig 1:**
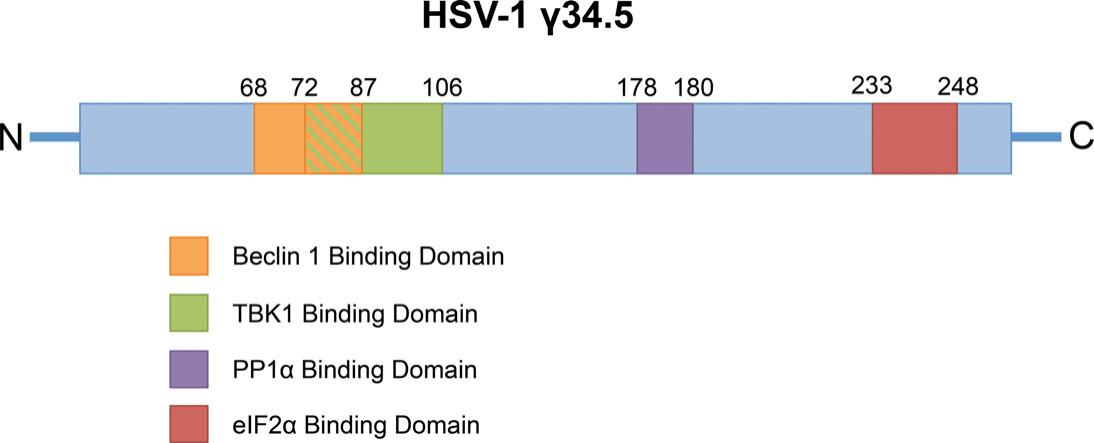
The HSV-1 major neurovirulence factor γ34.5 targets multiple different host pathways. The viral protein γ34.5 contains domains that specifically inhibit initiation of host autophagy through Beclin 1 binding, inhibit induction of the type I IFN response through TBK1 binding and also contains a C-terminal domain that retargets the host phosphatase PP1α to eIF2α for dephosphorylation and reversal of host cell-mediated translational arrest. The numbers above the protein schematic denote the amino acids responsible for binding the host factors.

Herpes simplex virus (HSV) is a ubiquitous human pathogen that causes a wide spectrum of disease, ranging from asymptomatic viral shedding to lethal encephalitis and disseminated disease [1,2]. These viruses belong to the neurotropic subfamily of α-herpesviruses, and after initial replication in epithelial cells, HSV enters sensory neurons to establish latency in neural ganglia. HSV can also progress to active lytic replication in the central nervous system, resulting in devastating encephalitis. To successfully replicate in the host nervous system, HSV encodes several viral proteins to counter the host innate response to infection. Among these, the multifunctional viral protein γ34.5 is central to countering several effector pathways in the host type I interferon (IFN) response. HSV γ34.5 is present in two copies in the repeated regions of the viral genome, and although initially described as a late gene, its expression is actually “leaky late,” with γ34.5 functioning to counter the host response after late viral DNA synthesis but also in the first hours of infection. Within γ34.5 are domains that specifically target host shutoff of protein synthesis [3], type I IFN induction through TANK-binding kinase (TBK1) [4], and inhibition of autophagy through Beclin 1 binding (Fig 1) [5]. HSV γ34.5 is required for full virulence in the murine brain [6,7]; however, recent evidence suggests that γ34.5 may function differently in newborn models of HSV disease compared to the adult [8]. Furthermore, some functions of γ34.5 are required for pathogenesis in non-nervous system tissue [9]. Here, we provide a brief overview of the multiple host responses modulated by γ34.5 for successful HSV replication in the nervous system and also discuss recent evidence that expands the role of γ34.5 to promote pathogenesis in several different tissue-types and across different developmental ages of the host.

## HSV-1 γ34.5 Mediates Reversal of Host Shutoff of Total Protein Synthesis

**Fig 2:**
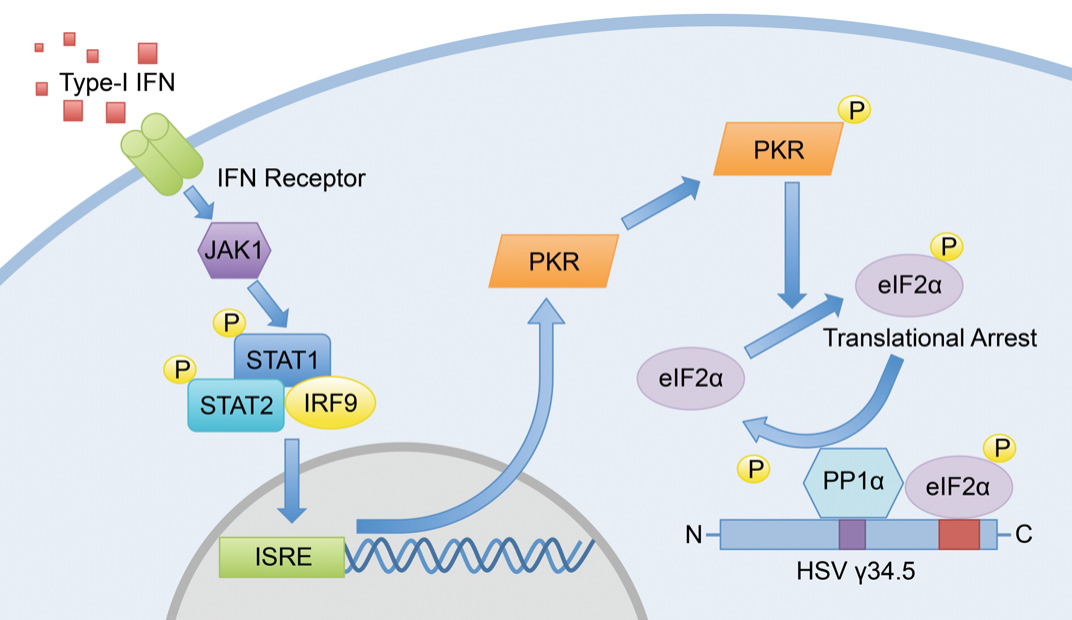
Reversal of the host shutoff of protein synthesis mediated by HSV γ34.5. During viral infection, the host cell detects type I IFNs through the IFN receptor, activating the JAK-STAT pathway and up-regulating several interferon-stimulated genes (ISGs), one of which is the kinase PKR. Once activated by one of its ligands (dsRNA or PACT), a major function of PKR is to phosphorylate the host translation initiation factor eIF2α to cause translational arrest and global inhibition of both viral and host protein synthesis. However, the HSV γ34.5 protein binds the host phosphatase PP1α and retargets it to eIF2α for dephosphorylation and restoration of mRNA translation. Viruses mutant in only the two amino acids required for PP1α-binding are significantly attenuated for disease in models of encephalitis, disseminated disease, and HSV keratitis.

One of the earliest responses to infection is the type I IFN response and the innate pathways modulated by the IFN-inducible, double-stranded RNA–dependent protein kinase R (PKR) system. An important function of activated PKR during HSV infection is phosphorylation of the translation initiation factor eIF2α, resulting in translational arrest and reduction in the global synthesis of viral and cellular proteins [10]. However, HSV has evolved an effective strategy through γ34.5 to reverse the eIF2α kinase-mediated translational arrest to allow for successful viral replication. The carboxyl terminus of HSV-1 γ34.5 binds and retargets the host phosphatase PP1α to eIF2α, thus targeting eIF2α for dephosphorylation and reversing the shutoff of protein synthesis (Fig 2) [11]. Mutant viruses engineered to specifically disrupt the interaction between γ34.5 and the host phosphatase PP1α demonstrate the requirement of HSV-1–mediated retargeting of PP1α for pathogenesis in several different models of disease, including HSV keratitis [12], encephalitis, and disseminated disease in the neonate [9]. Interestingly, the carboxyl terminus of HSV-1 γ34.5 shares sequence homology with the host protein GADD34 (growth arrest and DNA damage-inducible gene 34) [13], which acts as PP1α regulatory subunit to target PP1α to eIF2α during periods of endoplasmic reticulum (ER) stress and the unfolded protein response. Earlier studies have shown that this host sequence and γ34.5 are interchangeable in the HSV-1 genome to preclude the premature shutoff of total protein synthesis, suggesting that during herpesvirus evolution, the virus acquired the GADD34 host sequence to improve viral replication and fitness [14].

## γ34.5 Binds TBK1 to Prevent Activation of the Type I IFN Response

Prior to the initiation of the type I IFN response, HSV is detected in the host cell through several different pattern recognition receptors. For example, Toll-like receptor 3 (TLR3) detects HSV dsRNA in endosomes to stimulate IFN expression. In the cytoplasm, intracellular RNA and DNA sensors, such as retinoic acid-inducible gene I (RIG-I), melanoma differentiation-associated gene 5 (MDA5), interferon γ-inducible protein 16 (IFI16), and cyclic GMP-AMP synthase (cGAS), also detect HSV in the host cell [15–17]. Although these receptors detect different pathogen-associated molecular patterns, downstream signals are relayed through TBK1, which in turn phosphorylates and activates the interferon regulatory factor 3/7 (IRF3/7) for production of type I IFNs. HSV-1 γ34.5 counters this induction of the type I IFN response through binding of TBK1 with its amino terminus (Fig 1) [4]. Targeting of TBK1 by γ34.5 competes for IRF3 binding and ultimately inhibits IRF3 phosphorylation by TBK1, preventing IRF3 nuclear localization for type I IFN expression. A mutant virus deleted for the amino terminus of γ34.5 to demolish TBK1 binding demonstrates significantly increased IFN-β and interferon-stimulated gene (ISG) production in the first three to six hours of infection. In an ocular model of HSV disease, a virus deleted for TBK1 binding replicated poorly in the corneal epithelium and trigeminal ganglion and was effectively controlled by the host response before it reached the brain [18]. These findings reveal an additional role for γ34.5 in inhibiting the host response prior to transcription of type I IFNs and PKR up-regulation and demonstrate a role for early expression of this “leaky-late” gene.

## γ34.5 Inhibits Host Autophagy through Beclin 1 Binding

Autophagy, the cellular process by which intracellular pathogens and proteins are degraded in a double-membraned autophagosome, is critical for the control of several neurotropic viruses, including HSV-1 [19,20]. In addition to direct lysosomal fusion and degradation of virions, autophagy plays a critical role in immune signaling, including antigen processing for MHC presentation and delivery of viral nucleic acids to endosomal TLRs. Autophagy is thought to be a particularly important host mechanism to control viral replication in the nervous system in order to prevent a cytolytic response in neurons, which could be very detrimental to the vertebrate host. Type I IFN signaling up-regulates PKR expression, which in turn can be activated by one of its activator ligands to induce autophagy during neuronal infection. In order to successfully replicate in the brain, HSV-1 γ34.5 binds and inhibits the autophagy-inducing protein Beclin 1 (Fig 1) [5], which is downstream of activated PKR. Mutant viruses deleted specifically for the Beclin 1-interacting domain of γ34.5 demonstrate robust activation of autophagy and significant reduction in viral replication in vitro and in vivo. In comparison, wild-type HSV-1 γ34.5 is very effective at inhibiting autophagy and can even suppress autophagy below basal levels in the host cell. In addition to the innate immune response to infection, autophagy plays a critical role in normal cell function, metabolism, and development. Importantly, autophagy is required for proper neurodevelopment and is rapidly up-regulated after birth in the newborn in the early neonatal starvation period. This unique autophagic environment in the newborn brain may explain the surprising recent finding that inhibition of autophagy by HSV-1 γ34.5 is dispensable for pathogenesis in this age group, and wild-type HSV-1 is unable to effectively suppress autophagy in the newborn brain [8]. Studying the autophagy-inhibiting function of the HSV protein γ34.5 has not only helped understand how the virus successfully targets the host response to replicate in neurons but also provides significant insight into the mechanisms of the host response and how they might differ between different developmental ages.

## The Structure and Function of γ34.5 Differs Significantly between HSV-1 and HSV-2

Although herpes simplex virus type 1 and type 2 are closely related neurotropic herpesviruses with colinear genomes, there are clear differences between the two viruses in terms of pathogenesis. In several different experimental animal models of disease, HSV-2 is more neurovirulent than HSV-1. While both viruses contain two copies of the γ34.5 gene located within the inverted repeat regions of the genome, recent evidence demonstrates significant differences in the γ34.5 sequence and expression between the two HSV serotypes. In contrast to the HSV-1 homologue, the HSV-2 major neurovirulence factor γ34.5 is a spliced gene that contains an intron [21]. Furthermore, it was recently shown that unlike HSV-1, there are up to four distinct polypeptides produced from the open reading frame of HSV-2 γ34.5 [22]. Sequence alignment between the two full-length proteins reveals significant amino acid conservation in the C-terminal region, which is responsible for targeting host-mediated translational arrest. However, the N-terminal domain in HSV-1 γ34.5, responsible for binding Beclin 1 and TBK1, shares only some sequence homology with HSV-2 γ34.5, with insertions appearing to disrupt the corresponding Beclin 1 and TBK1 domains in HSV-2. Although the reversal of host cell-mediated translational arrest by γ34.5 is conserved between HSV serotypes [23], it is likely that there are additional undescribed functions of HSV-2 γ34.5 and the different peptide forms of HSV-2 γ34.5 that may contribute, at least in part, to differences in neuropathogenesis between the two viruses.

## Herpes Simplex Viruses Mutant in γ34.5 Are Used as Oncolytic Vectors

Oncolytic virotherapy employs lytic viruses to infect, replicate into, and ultimately kill cancer cells. Herpes simplex viruses are particularly well suited for this task because of their high seroprevelence in the general population, manipulable genome, and the ability to control replication with the antiviral acyclovir. One of the first HSV recombinants engineered for oncolytic therapy was deleted in the neurovirulence gene γ34.5 [6,24]. Because of its role in countering the IFN-mediated PKR response, deletion of γ34.5 resulted in conditional replication of oncolytic viruses in tumor cells that have low PKR activity, such as human glioma cells [24]. Interestingly, the differential replication and efficacy of γ34.5-mutant oncolytic viruses led to the discovery of heterogeneity in important innate immune pathways in the host cancer cell. It was found that PKR and its inhibitor MAPK/ERK kinase (MEK) have differential activity dependent on cell type and that some tumor cells have low MEK expression and thus poor replication of γ34.5-mutant viruses [25]. Although several different oncolytic virus strategies have been investigated since the first tumor-selective, γ34.5-mutant HSVs, the γ34.5-null viral vectors have completed Phase I and II trials and remain the most investigated vectors in current clinical trials [26–28].

## Perspectives

The HSV major neurovirulence factor γ34.5 was initially described over two decades ago, but the specific virus–host interactions and mechanisms of pathogenesis mediated by this multifunctional protein are still being elucidated. The γ34.5 protein provides an excellent example of how viruses have evolved to modulate a multitude of host immune responses with a very limited genome size and, in the case of reversal of host-mediated translational arrest, sometimes possibly adopt host functions during virus evolution. Investigations of γ34.5 have not only helped to understand how HSV has become such a successful pathogen but also provide insight into innate host responses such as autophagy, which has recently been described as a common strategy for controlling several different neurotropic viruses and bacteria. The unique expression pattern of γ34.5 throughout the viral life cycle has improved our understanding of the timing of host responses, such as type I IFN induction through TBK1 and reliance on PKR for Beclin 1 targeting by HSV-1. Interestingly, it was recently shown that the virus itself targets γ34.5 expression through the production of a viral miRNA (miR-I), expressed from the latency associate transcript (LAT) exon 2 [29]. miR-I was abundantly detected in latently infected trigeminal ganglia and was shown to specifically reduce γ34.5 expression. Furthermore, miRNAs produced from the LAT region and specifically targeted to γ34.5 were conserved between HSV serotypes. Tight regulation of γ34.5 by the virus itself through these viral miRNAs late in infection may be important for initiation of latency [29] and could represent a switch to allow for suppression of HSV replication by the host cell. The process of studying different γ34.5 functions has yielded several mutant viruses deleted for specific interactions with host proteins, and these mutants allow us to probe the host response across several different tissue-types and developmental ages. This has greatly improved our ability to investigate the host pathways that may dramatically contribute to disease severity after viral infection in the central nervous system and the exceedingly susceptible newborn host.

## References

[1] Pinninti SG, Kimberlin DW (2013) Neonatal herpes simplex virus infections. Pediatr Clin North Am 60: 351–365. 10.1016/j.pcl.2012.12.005 23481105

[2] Langenberg A, Corey L, Ashley R, Leong W, Straus S (1999) A prospective study of new infections with herpes simplex virus type 1 and type 2. Chiron HSV Vaccine Study Group. N Engl J Med 341: 1432–1438. 10547406 10.1056/NEJM199911043411904

[3] He B, Gross M, Roizman B (1997) The gamma(1)34.5 protein of herpes simplex virus 1 complexes with protein phosphatase 1alpha to dephosphorylate the alpha subunit of the eukaryotic translation initiation factor 2 and preclude the shutoff of protein synthesis by double-stranded RNA-activated protein kinase. Proc Natl Acad Sci U S A 94: 843–848. 9023344 10.1073/pnas.94.3.843 PMC19601

[4] Verpooten D, Ma Y, Hou S, Yan Z, He B (2009) Control of TANK-binding kinase 1-mediated signaling by the gamma(1)34.5 protein of herpes simplex virus 1. J Biol Chem 284: 1097–1105. 10.1074/jbc.M805905200 19010780 PMC2613634

[5] Orvedahl A, Alexander D, Talloczy Z, Sun Q, Wei Y, et al (2007) HSV-1 ICP34.5 confers neurovirulence by targeting the Beclin 1 autophagy protein. Cell Host Microbe 1: 23–35. 18005679 10.1016/j.chom.2006.12.001

[6] Chou J, Kern ER, Whitley RJ, Roizman B (1990) Mapping of herpes simplex virus-1 neurovirulence to gamma 134.5, a gene nonessential for growth in culture. Science 250: 1262–1266. 2173860 10.1126/science.2173860

[7] Bolovan CA, Sawtell NM, Thompson RL (1994) ICP34.5 mutants of herpes simplex virus type 1 strain 17syn+ are attenuated for neurovirulence in mice and for replication in confluent primary mouse embryo cell cultures. J Virol 68: 48–55. 8254758 10.1128/jvi.68.1.48-55.1994 PMC236262

[8] Wilcox DR, Wadhwani NR, Longnecker R, Muller WJ (2015) Differential reliance on autophagy for protection from HSV encephalitis between newborns and adults. PLoS Pathog 11: e1004580 10.1371/journal.ppat.1004580 25569138 PMC4287605

[9] Wilcox DR, Muller WJ, Longnecker R (2015) HSV targeting of the host phosphatase PP1α is required for disseminated disease in the neonate and contributes to pathogenesis in the brain. Proceedings of the National Academy of Sciences USA 112: E6937–44. 10.1073/pnas.1513045112 PMC4687582 26621722

[10] Gale M Jr., Katze MG (1998) Molecular mechanisms of interferon resistance mediated by viral-directed inhibition of PKR, the interferon-induced protein kinase. Pharmacol Ther 78: 29–46. 9593328 10.1016/s0163-7258(97)00165-4

[11] Cheng G, Gross M, Brett ME, He B (2001) AlaArg motif in the carboxyl terminus of the gamma(1)34.5 protein of herpes simplex virus type 1 is required for the formation of a high-molecular-weight complex that dephosphorylates eIF-2alpha. J Virol 75: 3666–3674. 11264356 10.1128/JVI.75.8.3666-3674.2001 PMC114858

[12] Verpooten D, Feng Z, Valyi-Nagy T, Ma Y, Jin H, et al (2009) Dephosphorylation of eIF2alpha mediated by the gamma134.5 protein of herpes simplex virus 1 facilitates viral neuroinvasion. J Virol 83: 12626–12630. 10.1128/JVI.01431-09 19759130 PMC2786747

[13] McGeoch DJ, Barnett BC (1991) Neurovirulence factor. Nature 353: 609. 10.1038/353609b0 1656275

[14] He B, Chou J, Liebermann DA, Hoffman B, Roizman B (1996) The carboxyl terminus of the murine MyD116 gene substitutes for the corresponding domain of the gamma(1)34.5 gene of herpes simplex virus to preclude the premature shutoff of total protein synthesis in infected human cells. J Virol 70: 84–90. 8523596 10.1128/jvi.70.1.84-90.1996 PMC189791

[15] Rasmussen SB, Jensen SB, Nielsen C, Quartin E, Kato H, et al (2009) Herpes simplex virus infection is sensed by both Toll-like receptors and retinoic acid-inducible gene- like receptors, which synergize to induce type I interferon production. J Gen Virol 90: 74–78. 10.1099/vir.0.005389-0 19088275 PMC2956989

[16] Li XD, Wu J, Gao D, Wang H, Sun L, et al (2013) Pivotal roles of cGAS-cGAMP signaling in antiviral defense and immune adjuvant effects. Science 341: 1390–1394. 10.1126/science.1244040 23989956 PMC3863637

[17] Thompson MR, Sharma S, Atianand M, Jensen SB, Carpenter S, et al (2014) Interferon gamma-inducible protein (IFI) 16 transcriptionally regulates type i interferons and other interferon-stimulated genes and controls the interferon response to both DNA and RNA viruses. J Biol Chem 289: 23568–23581. 10.1074/jbc.M114.554147 25002588 PMC4156042

[18] Ma Y, Jin H, Valyi-Nagy T, Cao Y, Yan Z, et al (2012) Inhibition of TANK binding kinase 1 by herpes simplex virus 1 facilitates productive infection. J Virol 86: 2188–2196. 10.1128/JVI.05376-11 22171259 PMC3302378

[19] Yordy B, Iijima N, Huttner A, Leib D, Iwasaki A (2012) A neuron-specific role for autophagy in antiviral defense against herpes simplex virus. Cell Host Microbe 12: 334–345. 10.1016/j.chom.2012.07.013 22980330 PMC3454454

[20] Orvedahl A, MacPherson S, Sumpter R Jr., Talloczy Z, Zou Z, et al (2010) Autophagy protects against Sindbis virus infection of the central nervous system. Cell Host Microbe 7: 115–127. 10.1016/j.chom.2010.01.007 20159618 PMC2860265

[21] Tang S, Guo N, Patel A, Krause PR (2013) Herpes simplex virus 2 expresses a novel form of ICP34.5, a major viral neurovirulence factor, through regulated alternative splicing. J Virol 87: 5820–5830. 10.1128/JVI.03500-12 23487469 PMC3648131

[22] Korom M, Davis KL, Morrison LA (2014) Up to four distinct polypeptides are produced from the gamma34.5 open reading frame of herpes simplex virus 2. J Virol 88: 11284–11296. 10.1128/JVI.01284-14 25031346 PMC4178799

[23] Davis KL, Korom M, Morrison LA (2014) Herpes simplex virus 2 ICP34.5 confers neurovirulence by regulating the type I interferon response. Virology 468–470: 330–339. 10.1016/j.virol.2014.08.015 25238641

[24] Andreansky SS, He B, Gillespie GY, Soroceanu L, Markert J, et al (1996) The application of genetically engineered herpes simplex viruses to the treatment of experimental brain tumors. Proc Natl Acad Sci U S A 93: 11313–11318. 8876132 10.1073/pnas.93.21.11313 PMC38054

[25] Smith KD, Mezhir JJ, Bickenbach K, Veerapong J, Charron J, et al (2006) Activated MEK suppresses activation of PKR and enables efficient replication and in vivo oncolysis by Deltagamma(1)34.5 mutants of herpes simplex virus 1. J Virol 80: 1110–1120. 16414988 10.1128/JVI.80.3.1110-1120.2006 PMC1346955

[26] Rampling R, Cruickshank G, Papanastassiou V, Nicoll J, Hadley D, et al (2000) Toxicity evaluation of replication-competent herpes simplex virus (ICP 34.5 null mutant 1716) in patients with recurrent malignant glioma. Gene Ther 7: 859–866. 10845724 10.1038/sj.gt.3301184

[27] Mace AT, Ganly I, Soutar DS, Brown SM (2008) Potential for efficacy of the oncolytic Herpes simplex virus 1716 in patients with oral squamous cell carcinoma. Head Neck 30: 1045–1051. 10.1002/hed.20840 18615711

[28] MacKie RM, Stewart B, Brown SM (2001) Intralesional injection of herpes simplex virus 1716 in metastatic melanoma. Lancet 357: 525–526. 11229673 10.1016/S0140-6736(00)04048-4

[29] Tang S, Bertke AS, Patel A, Wang K, Cohen JI, et al (2008) An acutely and latently expressed herpes simplex virus 2 viral microRNA inhibits expression of ICP34.5, a viral neurovirulence factor. Proc Natl Acad Sci U S A 105: 10931–10936. 10.1073/pnas.0801845105 18678906 PMC2504787

